# Influence of cerium oxide nanoparticles on dairy effluent nitrate and phosphate bioremediation

**DOI:** 10.1007/s10661-022-10003-0

**Published:** 2022-04-05

**Authors:** Abeer M. Salama, Moktar S. Behaery, Amira E. Abd Elaal, Ahmed Abdelaal

**Affiliations:** Environmental Sciences Department, Faculty of Science, Port Said University, Port Said, 42526 Egypt

**Keywords:** Cerium oxide nanoparticles, Nitrate, Phosphate, Activated sludge, Bacterial wastewater treatment, Bioremediation

## Abstract

This study investigated, for the first time, the role of cerium oxide nanoparticles (CeO_2_ NPs) on dairy effluent nitrate and phosphate bioremediation using different inoculum sources. Two inoculum sources (wastewater and sludge) were obtained from the dairy wastewater treatment plant unit. A culture was prepared to be tested in the treatment of nitrate and phosphate effluent, and the role of CeO_2_ NPs was checked to be completely efficient after 5 days of incubation. The reduction efficiency of nitrate using sludge as inoculum source was improved up to 89.01% and 68.12% for phosphate compared to control. In the case of using wastewater as an inoculum source, the nitrate reduction was improved up to 83.30% and 87.75% for phosphate compared to control. The bacterial richness showed a significant variance (higher richness) between control and other samples. The optimal concentration of CeO_2_ NPs for inoculum richness and nitrate and phosphate reduction was (sludge: 1 × 10^−10^ ppm) and (wastewater: 1 × 10^−12^ ppm). The results revealed that CeO_2_ NPs could enhance the microbial growth of different inoculum sources that have a key role in dairy effluent nitrate and phosphate bioremediation.

**Supplementary information:**

The online version contains supplementary material available at 10.1007/s10661-022-10003-0.

## Introduction

To date, there is a great challenge in the drivers for water change such as population growth, economic development, social, and technological change. These drivers have adversely negative impacts on water resources and climate change (Bassem, 2020). The unsustainable industrial development causes negative pressures on the environment. Industrial wastewater with high concentrations of pollutants (e.g., nitrate and phosphate) adversely affects the environment (Amoatey & Baawain, 2019). Declining the water quality in the countries with a scarcity of water resources reduces the country’s opportunity for sustainable industrial development and threatens public health with spreading infectious diseases (PAHO, 2013).

Nanoparticles (NPs) were investigated for various processes of wastewater treatment. The NP properties (e.g., high surface-to-volume ratios and reduced size) enable them to be highly reactive with distinct characteristics (Singh et al., 2019). Das et al. (2020) reported that NPs are highly effective in wastewater pollutants removal and are considered a promising method for wastewater treatment. Nano-bioremediation achievements flourished the technologies of wastewater treatment (Khan et al., 2019; Singh et al., 2020a, b).

Antioxidant nanomaterials such as cerium oxide (CeO_2_) NPs had recently obtained good attention for their massive potential in biotechnology (Casals et al., 2020). CeO_2_ NPs were used as catalysts, physicochemical burnish mediators, coverings, and fuel additives (Hu et al., 2018). CeO_2_ NPs also obtained a lot of attention in solving many problems through displaying redox action, and biofilm restraint, etc. (Nyoka et al., 2020; Singh et al., 2020a, b).

The formation of CeO_2_ nanoparticles was characterized using transmission electron microscopy (TEM) which used to assess the size and detailed morphology of the CeO_2_ NPs, X-ray diffraction (XRD), and zeta potential which applied to recognize the surface charge of CeO_2_ NPs and to study the stability of nanoparticles. Ultraviolet-visible (UV-vis) spectroscopy was used for the visual observation of the NPs formation, and Fourier transform infrared spectroscopy (FT-IR) was applied to determine the existence of specific surface functional groups in the investigated NPs. The crystalline phase analysis using X-ray diffraction exposed the amorphous nature of CeO_2_ NPs (Al-Ananzeh, 2021; García et al., 2012; Prabhakar et al., 2017).

CeO_2_ NPs were effective in the removal of different pollutants from the wastewater (Contreras et al., 2015). CeO_2_ NPs could improve the growth of some bacterial species that are shared in the bioremediation process. High concentrations of CeO_2_ NPs could have negative effects on the bioremediation process of phosphate removal using an activated sludge (Kamika & Tekere, 2017).

Different industrial wastewater pollutants, particularly with high nitrate and phosphate concentrations, were successfully removed using the biological treatment processes. The wastewater treatment processes were highly developed to achieve this task with low input of energy (Ahammad et al., 2013; Iloms et al., 2020). The efficiency of these processes can be referred to as the presence of key microorganisms in wastewater and activated sludge (Achmadulina et al., 2017).

High concentrations of nitrate perform adversely in oxygen transport procedures, leading to the hypoxia process and many human health problems (Cheng & Chen, 2001, 2002; Dutra et al., 2020; Luo et al., 2020). In intensive systems, aquatic organisms could be exposed to high nitrate concentrations that could change the water quality and negatively affect the organism’s metabolism (de Farias Lima et al., 2020; Romano & Zeng, 2007, 2009, 2013). The nitrate bioaccumulation in aquatic organism’s tissue can cause undesirable problems in humans after consumption (Wolfe & Patz, 2002). In addition, the ingestion of highly accumulated nitrates led to emerging of carcinogens in the digestive system (de Farias Lima et al., 2020; Song et al., 2015).

Phosphates help in the blood oxidation in the biota and are involved in numerous biochemical procedures (Choi et al., 2020; Naushad et al., 2017; Wiemer, 2020). Despite phosphate is not poisonous, it is responsible for surface water eutrophication; therefore, remediation methods have been constantly investigated to eliminate it in aqueous environments (Luengo et al., 2017; McPherson et al., 2004). Chronic influences of phosphates such as expansion inhibition, reduced fertility, and gene expression were detected in aquatic organisms (Yuan et al., 2018).

This study aims to investigate the role of CeO_2_ NPs in the activation of microorganisms for the dairy effluent nitrate and phosphate bioremediation process. Specifically, the parameters such as the concentration of CeO_2_ NPs and their impact on bacterial growth and nitrate and phosphate reduction were investigated and discussed in this study.

## Materials and methods

### Inoculum sample collection

Fresh inoculum samples of dairy wastewater and activated sludge were collected from a dairy wastewater treatment plant at Jumasa, Egypt. The samples were stored at 4 °C to maintain their inoculum properties.

### Cerium oxide nanoparticles

A powder sample (5 mg) of NP CeO_2_ (within a size: ≤ 25 nm) was obtained from Sigma-Aldrich^®^ chemical company, Ontario, Canada, and used in this study.

### Experimental setup

The different inoculum source solutions (100 mL) were inoculated separately in a reactor including 300 mL of culture media (d-glucose anhydrate, 2.5 g/L; MgSO_4_·7H_2_O, 0.5 g/L and KNO_3_, 0.18 g/L; prepared in distilled water) (Kamika & Tekere, 2017). All the chemicals used in the experimental work were obtained from Sigma-Aldrich^®^ chemical company, Ontario, Canada. The inoculum sources were separately treated with different concentrations of CeO_2_ NPs to investigate their impact on the microbial species in wastewater treatment plants.

The concentrations of CeO_2_ NPs in the samples were adjusted to be 1 × 10^−8^, 1 × 10^−9^, 1 × 10^−10^, 1 × 10^−11^, 1 × 10^−12^, 1 × 10^−13^, 1 × 10^−14^, and 1 × 10^−15^ ppm, and the non-treated was used as a control. The concentrations’ adjustment was done after a pilot study aimed to obtain the bacterial growth enhancement CeO_2_ NPs start concentration using 1 × 10^−1^, 1 × 10^−2^, 1 × 10^−3^, 1 × 10^−4^, 1 × 10^−5^, 1 × 10^–6^, 1 × 10^–7^, and 1 × 10^−8^ ppm. Incubation condition for maximizing the bacterial growth was performed at 35 °C and pH 7.

X-ray diffraction analysis (XRD) and transmission electron microscope (TEM) were performed to determine the mineral composition and shape of the studied CeO_2_ NPs. Zeta potential analysis was performed to identify the surface charge of CeO_2_ NPs. These analyses and nano-specifications were carried out in Nano Science and Technology Institute at Kafrelsheikh University, Egypt. The aliquot samples were used to determine nitrate and phosphate concentrations (ppm) using ion chromatography (Thermo Scientific, Dionex ICS-1100) (Yi et al., 2020).

The microbial growth was measured at a wavelength of 450 nm (Domínguez et al., 2001; Mauerhofer et al., 2018), using Jenway Model 6800 Spectrophotometer. Triplicate tests were carried out, and the mean and change percentages to control were recorded.

#### X-ray diffraction analysis

The XRD analysis was used to show the nano-size and peaks of the CeO_2_ NPs to approve their crystalline structure and pattern (Arockia et al., 2019; Pillai et al., 2020; Almessiere et al., 2020; Aref & Salem, 2020). The XRD analysis was carried out in Nano Science and Technology Institute at Kafrelsheikh University, Egypt.

#### Transmission electron microscope

Transmission electron microscopy (TEM) was employed to show the size and morphological investigations of the NPs (Arockia et al., 2019; Aref & Salem, 2020; Pillai et al., 2020). The TEM analysis was carried out in Nano Science and Technology Institute at Kafrelsheikh University, Egypt.

#### Zeta potential analysis

Zeta potential analysis was used to identify the surface charge of CeO_2_ NPs and their physical stability in the aqueous solutions (Ding et al., 2018; Gaikwad et al., 2019; Jiang et al., 2009; Joseph & Singhvi, 2019; Selvamani, 2019). The particle sizes, ζ-potential, and polydispersity index (PDI) of nanoparticles had been determined through a Nano-ZS Zetasizer analyzer (Meng et al., 2020). The zeta analysis was carried out in Nano Science and Technology Institute at Kafrelsheikh University, Egypt.

#### Ultraviolet–visible spectroscopy

Ultraviolet–visible spectroscopy was used for the visual observation of the NP formation by monitoring the alterations in the solution color through incubation time (Arockia et al., 2019). The UV–Vis analysis using was carried out in Nano Science and Technology Institute at Kafrelsheikh University, Egypt, using JASCO NIR Spectrophotometer/ model: V-770.

#### Fourier transform infrared spectroscopy

FT-IR spectrophotometer was employed in this study to determine the existence of specific surface functional groups of the studied samples (Madubuonu et al., 2020; Pillai et al., 2020; Varadavenkatesan et al., 2020). The FT-IR analysis was carried out in Nano Science and Technology Institute at Kafrelsheikh University, Egypt, using Infrared Spectrum Origin Jasco: model, FT-IR 6800typeA.

## Results and discussion

### CeO_2_ nanoparticle characterization

#### X-ray diffraction analysis

**Fig. 1:**
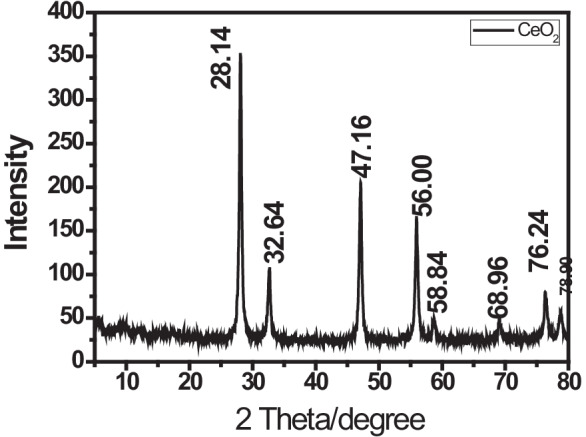
XRD pattern of CeO_2_ NPs

Figure 1 shows the XRD pattern of CeO_2_ NPs where well-defined peaks were obtained at 28.14°, 32.64°, 47.16°, 56.00°, 58.84°, 68.96°, 76.24°, and 78.90° corresponding to [346], [112], [218], [166], [38], [50], [76], and [50] planes of cubic CeO_2_ lattice. This diffraction pattern indicated that the NPs have very sharp peaks with ultrafine nature and high crystalline cubic spinel structure that confirm the purity and good formation of the metal-oxide NPs (Romer et al., 2019).

#### Transmission electron microscope

**Fig. 2:**
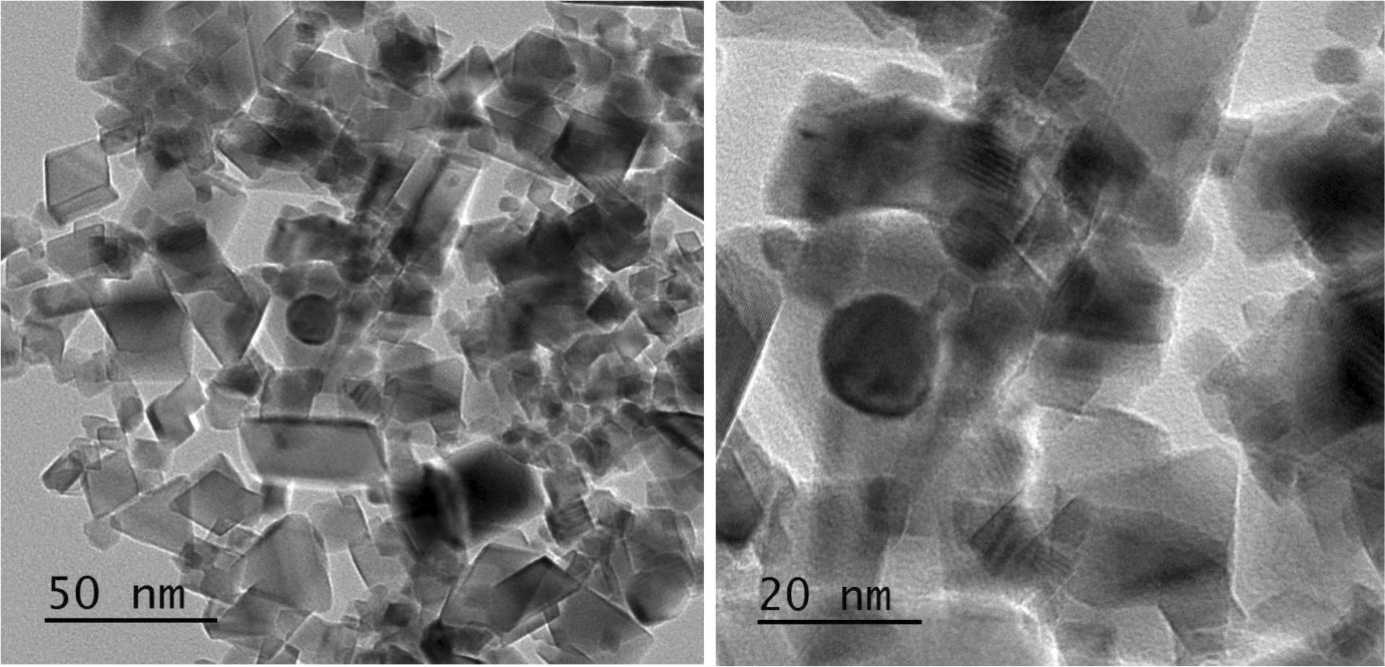
TEM photomicrograph of CeO_2_ NPs

TEM photomicrograph of the prepared CeO_2_ NPs is shown in Fig. 2 and indicates that the particles have an isotropic shape (Forest et al., 2017), within a range of 20–40 nm in size.

#### Zeta potential analysis

**Fig. 3:**
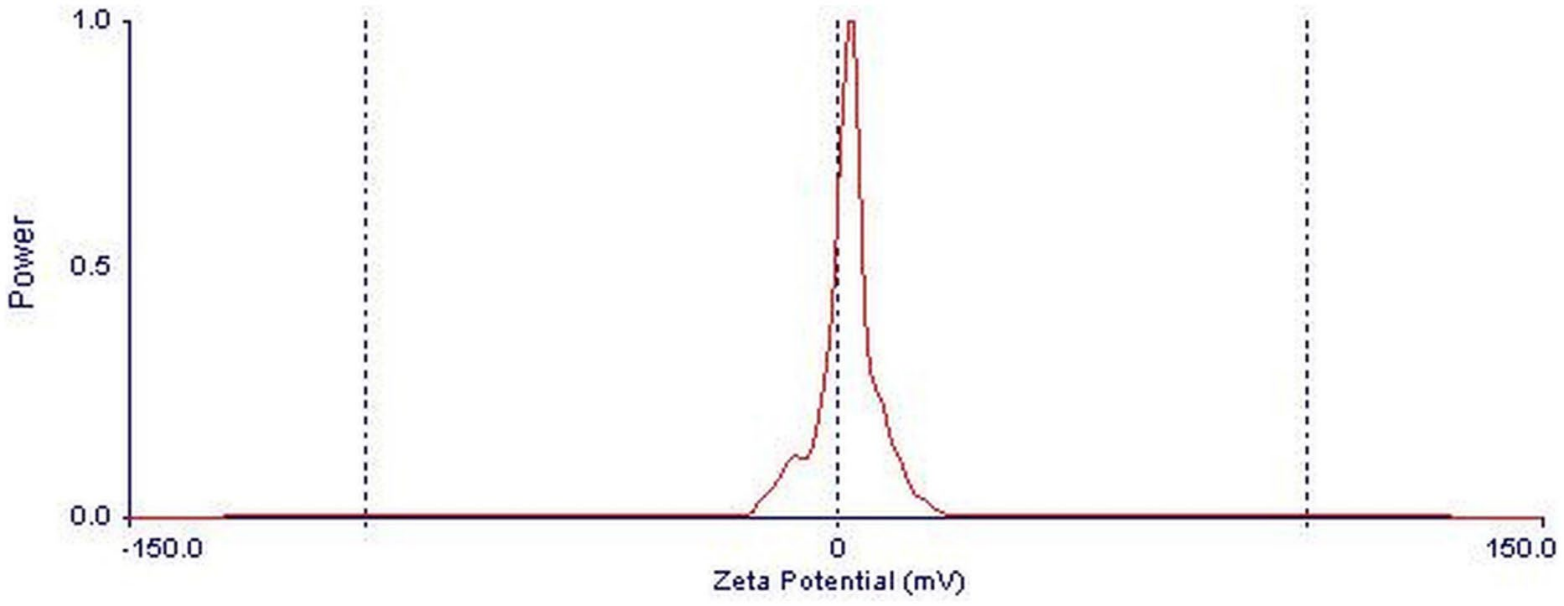
Zeta potential of CeO_2_ NPs

The zeta potential analysis of CeO_2_ NPs is shown in Fig. 3 indicating that the zeta potential value of CeO_2_ NPs is 1.5 mV and confirming its negative surface charge. Nanoparticles with zeta potential greater than + 25 mV or less than −25 mV have more colloidal stability with repulsive forces to avoid the agglomeration of NPs (Thakkar et al., 2016). Furthermore, the obtained results of CeO_2_ NPs indicated that the nanoparticles have a suitable dispersion capability in an aqueous medium.

#### Ultraviolet–visible spectroscopy

**Fig. 4:**
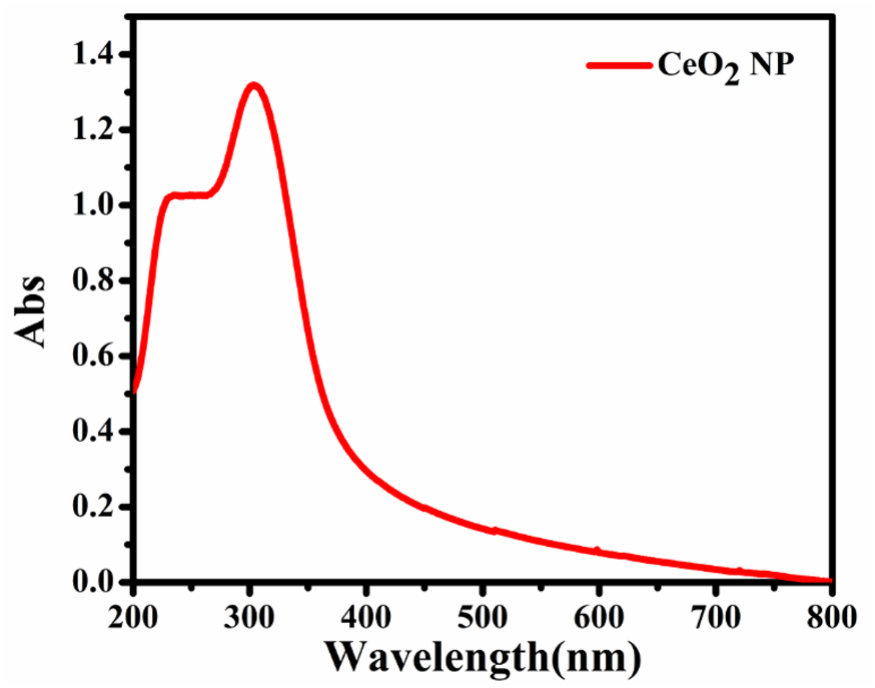
Ultraviolet–visible analysis of CeO_2_ NPs

Ultraviolet–visible analysis of CeO_2_ NPs is shown in Fig. 4 where the UV–Vis spectra at wavelengths of 200–800 nm were used to notice a powerful absorption peak, which related to superficial Plasmon excitation (Aref & Salem, 2020). The sharp peak assumed by the UV–Vis spectrum at the absorption wavelength is 340 nm (Fig. 4).

#### Fourier transform infrared spectroscopy

**Fig. 5:**
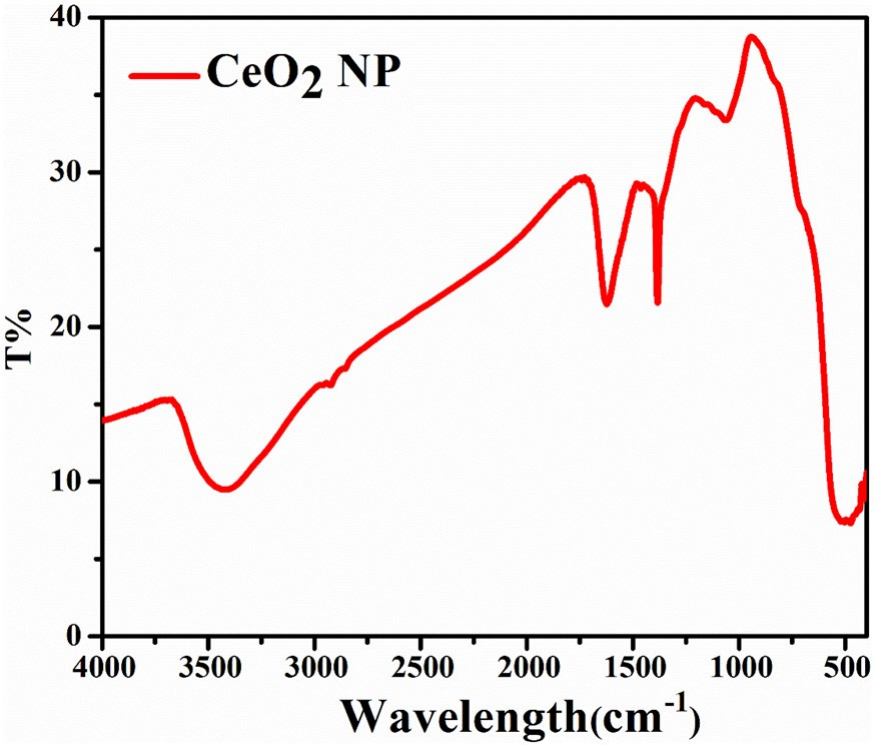
Fourier transform infrared (FT-IR) analysis of CeO_2_ NPs

Fourier transform infrared (FT-IR) analysis of CeO_2_ NPs in terms of wavenumber vs transmittance (%) is shown in Fig. 5. The evaluation was performed by using FT-IR spectrometer; the spectra were scanned in the wavelength range of 400–4000 Cm^−1^ at a resolution of 2 Cm^−1^ in KBr pellets (Aref & Salem, 2020; Sobhani-Nasab et al., 2020), where the maximum transmittance is 38.75% and the minimum transmittance is 7.31%.

### Bacteriological wastewater treatment

Microorganisms have a key role in pollutant degradation and biological systems maintenance and stabilization. Applying the technologies of biological wastewater treatment compared to other treatment actions has many advantages such as low cost, low or without secondary excretion of pollutants, and the most significant low adverse effects on the environment (Dadrasnia et al., 2017). Physico-chemical methods that were used for treating nitrate in wastewater (e.g., reverse osmosis (RO), electrodialysis, and ion exchange) generate secondary wastes which made these processes less desirable (Yun et al., 2016). In contrast, biological methods are more reliable and stable in wastewater treatment (McCarty, 2018). The presence of nitrate in polluted wastewater allows bacteria to obtain many metabolic capabilities enabling its adaptation to simulate nitrification-and denitrification processes (Rajta et al., 2020; Sharma & Dwivedi, 2017).

Numerous and diverse chemical, physico-chemical, and biological methods were used to remove phosphorus from wastewater. Chemical methods are less desirable due to their high cost and generate secondary pollution, while physico-chemical methods involve a high expenditure of the processes with a complex use. Furthermore, the biological methods of phosphorus removal are widely used worldwide (Ruzhitskaya & Gogina, 2017). There are series of technologies used for the biological removal of phosphorus such as phostrip, anaerobic/anoxic/oxic, activated sludge, and other technologies (Barnard, 2006).

The study results clearly explained the effectiveness of using microbial consortia (wastewater inoculum and sludge inoculum) in biological nitrogen and phosphorous remediation, which agree with the results of Wu et al. (2019), Zhang et al. (2019), Al Ali et al. (2020), Salama et al. (2022), Liu et al. (2017, 2018), Shomar et al. (2020), and Guemmaz et al. (2019) indicating the high efficiency of microbial consortia in simultaneous removal of nitrogen and phosphorous and particularly are more effective in bioremediation than using other pure microbial species.

### Effect of CeO_2_ nanoparticle concentrations on the bacterial growth

**Fig. 6:**
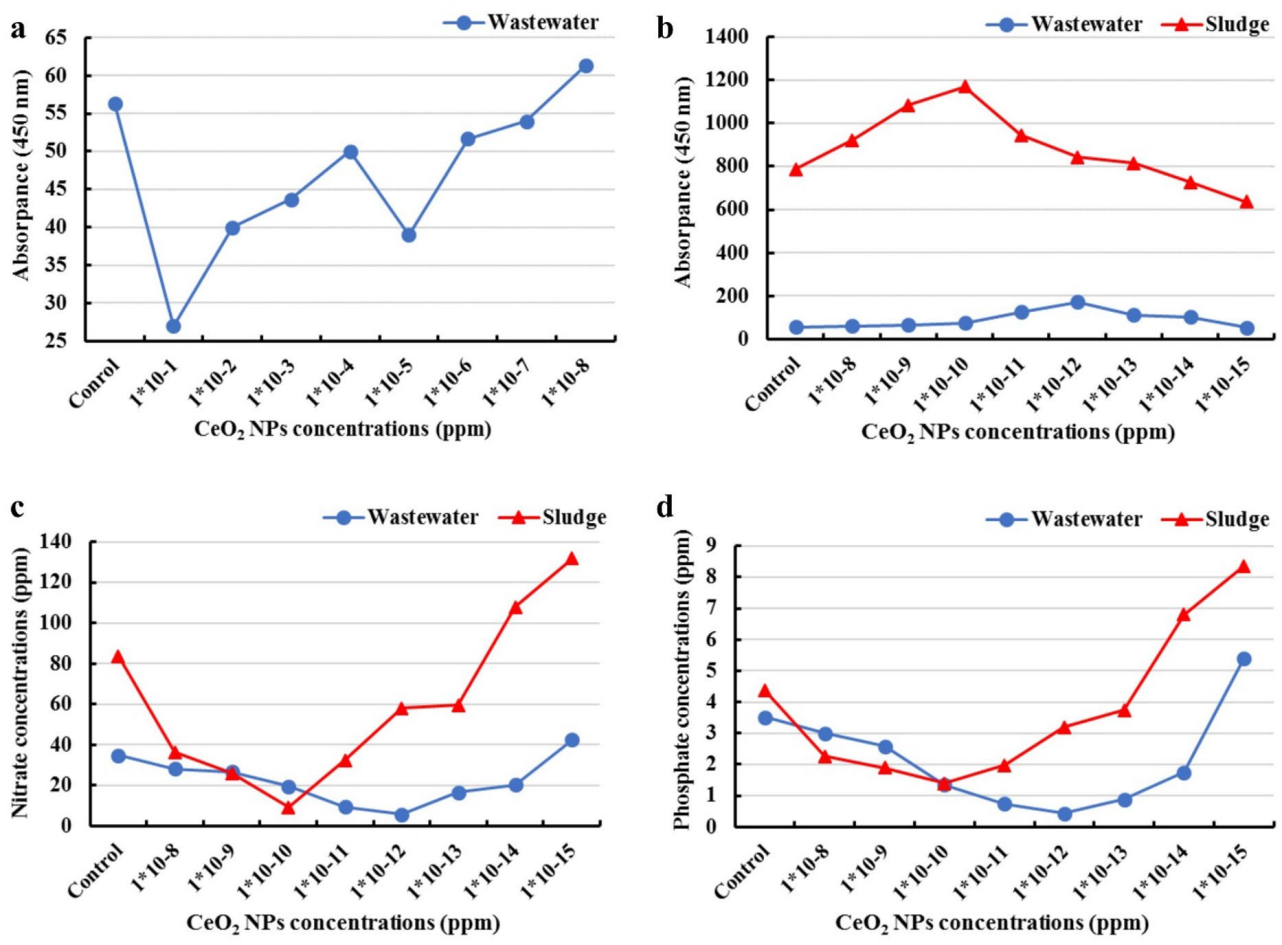
Absorbance pattern of microbial growth media at (450 nm) with wastewater as inoculum source (pilot study) **a** and using wastewater and sludge as inoculum source **b**. Reduction patterns of nitrate **c** and phosphate **d** concentrations (ppm) using different concentrations (ppm) of CeO_2_ NPs

The growth properties of wastewater inoculum were investigated using a spectrophotometer with CeO_2_ NP concentrations (from 1 × 10^−1^ to 1 × 10^−8^ ppm) that were used as a pilot study and were shown in Fig. 6a. While the growth properties of wastewater and sludge inoculum were investigated in presence of concentrations from (1 × 10^−8^ to 1 × 10^−15^ ppm) as shown in Fig. 6b.

Figure 6a, b show bi-phase dose–response relationships. A positive effect of CeO_2_ NPs on microbial growth was observed with a maximum of 1 × 10^−12^ ppm for wastewater inoculum and 1 × 10^−10^ ppm for sludge inoculum. However, a high-dose inhibition in biofilm formation was observed with CeO_2_ NPs higher than 1 × 10^−8^ ppm as shown in the pilot study’s results, which indicated the antimicrobial effects of CeO_2_ NPs. The statistical analysis of the studied data showed significant variations (*P* < 0.01) in the absorbance pattern of microbial growth media at 450 nm among different experimental factors (Table 1). These findings agree with those of Xu et al. (2019) indicating that the impacts of CeO_2_ NPs on microbial growth showed a typical effect, which was defined as a bi-phase dose–response relationship with low-dose stimulation and high-dose inhibition (Popov et al., 2017; Qiu et al., 2016; Salama et al., 2021; Xu et al., 2019).

**Table 1:** General linear model test for variation in absorbance pattern of bacterial growth media at 450 nm, using different concentrations (ppm) of CeO_2_ NPs

Growth media	Source	Degree of freedom	Sequential sums of squares	Adjusted sums of squares	Adjusted mean squares	*F*-value	*P*-value
Bacterial growth media + different concentrations of CeO_2_ NPs	Inoculum source	1	8,402,456	8,402,456	8,402,456	996.60	0.000
	Conc. of NPs (ppm)	8	354,214	354,214	44,277	5.25	0.000
	Error	44	370,971	370,971	8431	-	-
	Total	53	9,127,640	-	-	-	-

The low concentrations of CeO_2_ NPs improved the surface hydrophobicity, the aggregating ability in addition to the protein (PRO), and the polysaccharide (PS) microbial production during the initial attachment and differentiation process. The increased reactive oxygen species (ROS), which are produced by CeO_2_ NPs, promoted the production of the quorum sensing (QS) molecules by microbial organisms that resulting in the accelerated activation of QS systems (Xu et al., 2018). The QS among bacteria promotes the formation of biofilms, improves the strains’ resistance, promotes bacterial growth, and enhances the metabolic effects (Yang et al., 2020).

### Effect of CeO_2_ nanoparticle concentrations on dairy effluent nitrate and phosphate bioremediation

The bioremediation properties of dairy effluent were evaluated in terms of nitrate and phosphate examination. The role of different microbial inoculum sources on nitrate and phosphate decrease (ppm) was investigated using different concentrations of CeO_2_ NPs (from 1 × 10^−8^ to 1 × 10^−14^ ppm) for wastewater inoculum, and (from 1 × 10^−8^ to 1 × 10^−13^ ppm) for sludge inoculum (Fig. 6c, d).

By comparing the results obtained from the microbial growth with nitrate and phosphate reductions, it is noticed that the best microbial growth was at absorbance: 172.67 for wastewater inoculum, and 1170.33 for sludge inoculum, which coincides with the highest nitrate reduction (5.81 and 9.19 ppm) and highest phosphate reduction (0.43 and 1.39 ppm) that were achieved using (1 × 10^−12^ and 1 × 10^−10^ ppm) of CeO_2_ NPs, respectively, in the nutrient media (wastewater and sludge inoculum separately) that were compared to the control sample after 5 days of incubation at temperature 35 °C. Figure 6c, d show that nitrate and phosphate concentrations (ppm) linearly decrease with the increase of CeO_2_ NPs concentrations from 1 × 10^−8^ to 1 × 10^−14^ ppm for wastewater inoculum and from 1 × 10^−8^ to 1 × 10^−13^ ppm for sludge inoculum. A higher concentration of CeO_2_ NPs showed a lower bioremediation efficiency.

Statistical analysis of the data showed significant variations at *P* < 0.01 in nitrate and phosphate concentrations (ppm) between different experimental factors (microbial inoculum sources with different CeO_2_ NPs) (Tables 2 and 3).

**Table 2:** General linear model test for variation in nitrate (ppm) pattern, using different concentrations (ppm) of CeO_2_ NPs

Growth media	Source	Degree of freedom	Sequential sums of squares	Adjusted sums of squares	Adjusted mean squares	*F*-value	*P*-value
Bacterial growth media + different concentrations of CeO_2_ NPs	Inoculum source	1	19,399.5	19,399.5	19,399.5	54.04	0.000
	Conc. of NPs (ppm)	8	26,903.4	26,903.4	3362.9	9.37	0.000
	Error	44	15,794.9	15,794.9	359.0	-	-
	Total	53	62,097.7	-	-	-	-

**Table 3:** General linear model test for variation in phosphate (ppm) pattern, using different concentrations (ppm) of CeO_2_ NPs

Growth media	Source	Degree of freedom	Sequential sums of squares	Adjusted sums of squares	Adjusted mean squares	*F*-value	*P*-value
Bacterial growth media + different concentrations of CeO_2_ NPs	Inoculum source	1	34.10	34.10	34.10	31.86	0.000
	Conc. of NPs (ppm)	8	153.15	153.15	19.144	17.89	0.000
	Error	44	47.09	47.09	1.070	-	-
	Total	53	234.34	-	-	-	-

CeO_2_ NPs have delivered promising approaches in the bioremediation process. The physico-chemical properties of CeO_2_ NPs (e.g., size and surface charge) play key roles in the ultimate interactions of the nanoparticles with target cells (Charbgoo et al., 2017). Based on this, CeO_2_ NPs could enhance the metabolic activity of some microbial species while inhibiting those of others (Kamika & Tekere, 2017; Pelletier et al., 2010), depending on the enzymes that play a key role in the bacterial bioremediation (Jaiswal & Shukla, 2020).

Nitrification and denitrification are two major processes for biological nitrogen removal that organize the global nitrogen cycle. Four key enzymes carried out the denitrification process: nitrate reductase, nitrite reductase, nitric oxide reductase, and nitrous oxide reductase (Rajta et al., 2020). Furthermore, bacterial growth, which stimulates nitrate and phosphate removal enzymes, is a result of maximizing the bacterial count (Deng et al., 2020; Wang et al., 2020). According to Farias et al. (2018), the effect of CeO_2_ NPs on microbial count and activity is concentration dependent. The bacterial count and metabolic activity of some strains were enhanced by sub-lethal concentrations of CeO_2_ NPs exposure (Martínez et al., 2019; Xu et al., 2019).

The obtained results agree with those of Feng et al. (2019) and summarizing that exposure to higher concentrations of CeO_2_ NPs caused a sharp decrease in nitrogen and phosphorus removal efficiencies that were consistent with the tendencies of key enzymes (Feng et al., 2019). Specifically, CeO_2_ NPs at concentrations of 0.1, 1, and 10 ppm decreased the secretion of tightly bound extracellular polymeric substances to 0.13%, 3.14%, and 28.60%, respectively in comparison to the control. According to Wang et al. (2016), the removal rates of nitrate and phosphate show similar variation trends to the microbial enzymatic activities. Additionally, the variations of ROS and lactate dehydrogenase (LDH) indicated that a high concentration of CeO_2_ NPs could result in biotoxicity to the activated sludge (Wang et al., 2016). Overall, the high concentrations of CeO_2_ NPs could cause adverse effects on microbial richness and diversity of the activated sludge.

## Conclusions

Enhancing the reduction of nitrate and phosphate using bioremediation nanotechnologies is a major challenge. CeO_2_ NPs with sub-lethal concentrations have attracted interest due to their ability to produce higher bacterial growth, metabolic activity, and accordingly accelerate the nitrate and phosphate reduction. The bacterial growth together with nitrate and phosphate reduction were linearly correlated with the increase of CeO_2_ NP concentration. Nitrate and phosphate reduction’s efficiency, using sludge as an inoculum source, was improved up to 89.01% (for nitrate) and 68.12% (for phosphate) compared to control. In the case of using wastewater as an inoculum source, the nitrate and phosphate reduction was improved up to 83.30% and 87.75%, respectively, compared to control. The study findings concluded that using various inoculum sources together with the CeO_2_ NP concentrations is an efficient method for nitrate and phosphate reduction from dairy effluent.

## Supplementary Information

Below is the link to the electronic supplementary material.

Supplementary file1 (DOCX 25 KB)

## Data Availability

Fresh dairy wastewater and activated sludge inoculum samples were collected from a dairy wastewater treatment plant at Jumasa, Egypt. The dairy factory agreed to give different inoculum samples for this study. The permission’s letter was given without any obligation and commitment on our part.
