# Supplementary material for: Influence of cerium oxide nanoparticles on dairy effluent nitrate and phosphate bioremediation
Source: Environ Monit Assess. 2022 Apr 5;194(5):326. doi: 10.1007/s10661-022-10003-0 (PMC8983513; doi:10.1007/s10661-022-10003-0)
Supplement: Supplementary file 1 — Supplementary file1 (DOCX 25 KB) [file 10661_2022_10003_MOESM1_ESM.docx]

**Journal: Environmental Monitoring and Assessment**

**Influence of Cerium Oxide Nanoparticles on Dairy Effluent nitrate and phosphate biodegradation**

Abeer M. Salama^1^, Amira E. Abd Elaal^1^, Moktar S. Behaery^1^, Ahmed Abdelaal^1*^

^1^Environmental Sciences Department, Faculty of Science, Port Said University, 42526 Egypt

*Corresponding author: [ahmed_abdelaal@sci.psu.edu.eg](mailto:ahmed_abdelaal@sci.psu.edu.eg), ORCID: 0000-0002-7121-3674

**Supplementary Material**

**Table S1** The physico-chemical parameters of the diary waste water

| Physico-chemical parameters of dairy wastewater (Mean ± SD) | |
| --- | --- |
| pH | 7.3±0.1 |
| Biochemical oxygen demand (BOD) (mg/L) | 1450±150 |
| Chemical oxygen demand (COD) (mg/L) | 2666.67±152.75 |
| Total suspended solids (TSS) (mg/L) | 74066.67±1625.83 |
| Nitrate (mg/L) | 34.79 ± 1.68 |
| Phosphate (mg/L) | 3.51 ± 0.08 |

**Table S2** Mean (±SD) of replicates tests of the pilot study bacterial growth media absorbance at 450 nm and change (%) of absorbance than control, using different concentrations of CeO_2_ NPs (highest and lowest values are in bold)

| Growth media | Nanoparticles concentration (ppm) | Mean ± SD absorbance using wastewater inoculum  (450 nm) | Change % of absorbance |
| --- | --- | --- | --- |
| Different inoculum sources + CeO_2_ NPs | 0.00 | 56.33 ± 1.53 | 0.00 |
|  | 1*10^-1^ | **27.00 ± 2.00** | -52.1 |
|  | 1*10^-2^ | 40.00 ± 2.00 | -28.99 |
|  | 1*10^-3^ | 43.67 ± 0.58 | -22.47 |
|  | 1*10^-4^ | 50.00 ± 2.65 | -11.24 |
|  | 1*10^-5^ | 39.00 ± 1.00 | -30.77 |
|  | 1*10^-6^ | 51.67 ± 2.08 | -8.27 |
|  | 1*10^-7^ | 54.00 ± 4.00 | -4.14 |
|  | 1*10^-8^ | **61.33 ± 1.15** | 8.88 |

**Table S3** Mean (±SD) of replicates tests of bacterial growth media absorbance at 450 nm and change (%) of absorbance than control, using different concentrations of CeO_2_ NPs (highest and lowest values are in bold)

| Growth media | Nanoparticles concentration (ppm) | Mean ± SD absorbance using wastewater inoculum  (450 nm) | Change (%) of absorbance | Mean ± SD absorbance using sludge inoculum  (450 nm) | Change (%) of absorbance |
| --- | --- | --- | --- | --- | --- |
| Different inoculum sources + CeO_2_ NPs | 0.00 | 56.33 ± 1.53 | 0.00 | 786.67 ± 7.64 | 0.00 |
|  | 1*10^-8^ | 61.33 ± 1.51 | 8.88 | 920.33 ± 1.53 | 16.99 |
|  | 1*10^-9^ | 65.33 ± 1.53 | 15.79 | 1082.67 ± 17.50 | 36.63 |
|  | 1*10^-10^ | 75.33 ± 3.51 | 33.73 | **1170.33 ± 7.51** | 48.77 |
|  | 1*10^-11^ | 125.33 ± 0.58 | 123.21 | 944.00 ± 12.00 | 20.00 |
|  | 1*10^-12^ | **172.67 ± 3.79** | 206.51 | 843.67 ± 4.73 | 7.25 |
|  | 1*10^-13^ | 112.67 ± 3.06 | 100.00 | 815.33 ± 4.51 | 3.64 |
|  | 1*10^-14^ | 102.00 ± 1.00 | 82.25 | 726.33 ± 8.50 | -7.67 |
|  | 1*10^-15^ | **54.00 ± 2.00** | -4.14 | **636.00 ± 2.00** | -19.15 |

**Table S4** Mean (±SD) of nitrate concentration (ppm) and change (%) of reduction than control, using different concentrations of CeO_2_ NPs (highest and lowest values are in bold)

| Growth media | Nanoparticles concentration (ppm) | Mean ± SD nitrate reduction using wastewater inoculum  (450 nm) | Change (%) of reduction | Mean ± SD nitrate reduction using sludge inoculum  (450 nm) | Change (%) of reduction |
| --- | --- | --- | --- | --- | --- |
| Different inoculum sources + CeO_2_ NPs | 0.00 | 34.79 ± 1.68 | 0.00 | 83.58 ± 3.22 | 0.00 |
|  | 1*10^-8^ | 28.09 ± 0.43 | -19.26 | 36.26 ± 0.42 | -13.09 |
|  | 1*10^-9^ | 26.60 ± 0.28 | -23.54 | 26.04 ± 1.96 | -68.84 |
|  | 1*10^-10^ | 19.46 ± 0.14 | -44.06 | **9.19 ± 0.83** | -89.01 |
|  | 1*10^-11^ | 9.43 ± 0.43 | -72.89 | 32.48 ± 2.22 | -60.94 |
|  | 1*10^-12^ | **5.81 ± 0.21** | -83.30 | 58.00 ± 1.62 | -30.61 |
|  | 1*10^-13^ | 16.52 ± 1.12 | -52.53 | 59.41 ± 0.29 | -28.92 |
|  | 1*10^-14^ | 20.25 ± 0.80 | -41.79 | 107.91 ± 3.22 | 29.10 |
|  | 1*10^-15^ | **42.58 ± 2.29** | 22.36 | **131.69 ± 8.95** | 57.56 |

**Table S5.** Mean (±SD) of phosphate concentration (ppm) and change (%) of reduction than control, using different concentrations of CeO_2_ NPs (highest and lowest values are in bold).

| Growth media | Nanoparticles concentration (ppm) | Mean ± SD phosphate reduction using wastewater inoculum  (450 nm) | Change (%) of reduction | Mean ± SD phosphate reduction using sludge inoculum  (450 nm) | Change (%) of reduction |
| --- | --- | --- | --- | --- | --- |
| Different inoculum sources + CeO_2_ NPs | 0.00 | 3.51 ± 0.08 | 0.00 | 4.36± 00.07 | 0.00 |
|  | 1*10^-8^ | 3.00 ± 0.09 | -14.53 | 2.27 ± 00.14 | -47.95 |
|  | 1*10^-9^ | 2.58 ± 0.14 | -26.5 | 1.89 ± 0.01 | -56.65 |
|  | 1*10^-10^ | 1.35 ± 0.08 | -61.54 | **1.39 ± 0.17** | -68.12 |
|  | 1*10^-11^ | 0.75 ± 0.07 | -78.63 | 1.97 ± 0.06 | -54.82 |
|  | 1*10^-12^ | **0.43 ± 0.04** | -87.75 | 3.19 ± 0.08 | -26.83 |
|  | 1*10^-13^ | 0.92 ± 0.88 | -73.79 | 3.74 ± 0.11 | -14.22 |
|  | 1*10^-14^ | 1.75 ± 0.27 | -50.14 | 6.80 ± 0.45 | 55.96 |
|  | 1*10^-15^ | **5.41 ± 0.25** | 54.13 | **8.36 ± 0.48** | 91.74 |
